# Do We Need Premedication Before Coronary Angiography? A Controlled Clinical Trial

**DOI:** 10.4021/cr68w

**Published:** 2011-09-20

**Authors:** Hussein Alamri, Abdulrahman Almoghairi, Ali Almasood, Mohamed Alotaibi, Sami Alonazi

**Affiliations:** a Adult Cardiology Department, Prince Sultan Cardiac Center (PSCC), Riyadh, Saudi Arabia

**Keywords:** Premedication, Diazepam, Chlorpheniramine, Anxiety, Coronary angiography

## Abstract

**Background:**

Premedication with benzodiazepines has been thought to reduce patient anxiety, pain perception, and non-catheter-induced coronary spasms and may increase procedure-related complications. We used to routinely provide premedication with diazepam and chlorpheniramine before cardiac catheterization procedures. However the benefits of such a treatment are not well established here. Therefore, we designed this study to test whether the routine use of premedication during coronary angiography is needed.

**Methods:**

A total of 200 consecutive patients scheduled to undergo either diagnostic or therapeutic coronary angiographic procedures were randomized to receive either premedication with diazepam (5 mg) and chlorpheniramine (4 mg) 60 minutes prior to their procedures (n = 100) or no premedication (n = 100). The administration of intravenous midazolam during the procedures was permitted at the operator’s discretion. The primary endpoints were anxiety and pain perception during the procedure.

**Results:**

A total of 200 patients with similar baseline characteristics were randomized into two groups. The first group received oral premedication with diazepam (5 mg) and chlorphenamine (4 mg) 60 minutes prior to their procedures, and the other group did not receive premedication. We observed no differences in periprocedural pain perception (31% in the premedicated group versus 29% in the non-premedicated group; P = 0.75) or anxiety (59% in the premedicated group versus 50% in the non-premedicated group; P = 0.2). Interestingly, local pain was more pronounced in the premedicated patients than in the non-premedicated patients (30% versus 16%, respectively; P = 0.018). There were no contrast-related reactions reported in either group.

**Conclusion:**

Treatment with oral diazepam and chlorphenamine prior to cardiac catheterization and percutaneous coronary intervention does not alter rates of anxiety, periprocedural pain.

## Introduction

We used to routinely administer premedication with diazepam and chlorpheniramine to our patients undergoing cardiac catheterization procedures. We searched the literature for support for this approach, but the few relevant papers found only a neutral result when studying the effect of premedication on procedural outcomes [1]. Whether the cost of those medications outweighs their clinical benefits needs to be determined along with their effects on reducing procedural adverse events, patient pain perception, and anxiety. In a retrospective study, non-catheter-induced coronary spasms were observed in some of the patients for whom the sedation was the only premedication that was administered [2]. In contrast, a study of 12 patients who were administered intravenous diazepam before coronary angiography observed that, in addition to its central sedative effects, diazepam also has nitroglycerin-like effects on the coronary and systemic circulation [3]. Various sedatives have been used for premedication, but they are no longer routinely ordered to be administered before the patient is sent to the catheterization laboratory. Instead, the patient's state of alertness and need for sedation are assessed (if needed) once he or she is on the catheterization table. According to conscious sedation guidelines, the administration of small repeated doses of intravenous midazolam (0.5 to 1 mg) and/or fentanyl (25 to 50 mg) maintains a comfortable but arousable state [4]. Premedications have routinely been used in catheterization laboratories, but no evidence-based practices or previous studies have addressed their harmful or beneficial effects on procedural outcomes. In this study, we evaluated their usefulness and whether there is a difference between premedicating and non-premedicating practices. Doing so will add evidence-based practices (instead of inherited practices) to the literature.

## Materials and Methods

### Study design and patients

This randomized, single-center, controlled trial compared premedication with diazepam and chlorphenamine to no premedication in stable patients undergoing diagnostic or interventional procedures. A total of 200 patients were equally divided to receive or not receive open-label oral diazepam (5 mg) and chlorphenamine (4 mg) before cardiac catheterization.

This was a randomized, single-center, controlled clinical trial in which the patients were randomly divided equally into two groups (at a 1:1 ratio) and received either open-label oral diazepam (5 mg) and chlorphenamine (4 mg) or no premedication beforehand. The patients underwent either routine coronary angiography or PCI. This study only included adult patients who were referred for cardiac catheterization procedures. Any patients with acute myocardial infarction, decompensated heart failure or hemodynamic instability and any patients who were inappropriate due to high anxiety levels were excluded. The patients who were eligible for this clinical trial were given a detailed explanation of the study, and informed consent was obtained for the catheterization procedure. They were then randomized into one group that received premedication with diazepam (5 mg) 30 to 60 minutes prior to the procedures or a second group that did not receive the premedication.

Randomization was achieved using an “alternating weekdays” procedure. All of the patients who arrived for their procedures on Saturday, Monday and Wednesday were given the premedication, and the patients undergoing their procedures on Sunday and Tuesday received no premedication prior to the procedures.

It is also important to note that midazolam administration was permitted during the procedures and was performed at the operator’s discretion.

A data collection form was developed to collect the required information during recovery (while the patients were entering and exiting the catheterization laboratory). In addition, it took the current hospital practice and policy into account. This form included the patient’s demographic and background variables, including height and weight, comorbid conditions, and previous ischemic heart disease, which consisted of any previous history of ST-elevated myocardial infarction, non-ST-elevated myocardial infarction, or coronary artery bypass grafting. In addition, it documented which procedure they underwent (diagnostic or interventional cardiac catheterization) and whether the procedure was elective or acute. The primary endpoint was patient pain and anxiety. One of the aims of this study was to measure the number of adverse events that the patients experienced while undergoing the procedure. Therefore, this aspect of the research sought to assess adverse effects, such as the composite of contrast-related allergies. Secondary endpoints included the number of non-catheter-induced spasms. The patient’s anxiety and pain assessment involved a patient questionnaire, which sought to measure the patient’s self-assessment of their perception of pain and anxiety in the periprocedural period.

### Study medications

Diazepam (Valium) administration. Diazepam (5 mg) was administered within one hour of the procedure.

Chlorphenamine (Allerfin) administration. Chlorphenamine (4 mg) was administered within one hour of the procedure.

### Study endpoints

The primary endpoints included the effect of premedication on the patient’s anxiety and pain. The secondary endpoints included non-catheter-induced spasms and contrast-related allergies.

The study protocol was reviewed and approved by the institutional ethics and research committee at Prince Sultan Cardiac Center, Riyadh.

### Statistical analysis

Statistical analyses were performed using SPSS for Windows 16.0 (SPSS Inc., Chicago). The data are presented as the means ± standard deviations for the continuous variables and as absolute numbers (percentages) for the categorical variables. Comparisons between the two groups were performed using t-tests for the continuous variables and chi-squared tests for the categorical variables. A P-value of < 0.05 was considered to be statistically significant. An intention-to-treat analysis of all of the participants who were randomized into the treatment groups was used to determine the efficacy of the premedication therapy.

### Study limitations

This study is single-center study and may be subject to bias.

## Results

**Figure 1:**
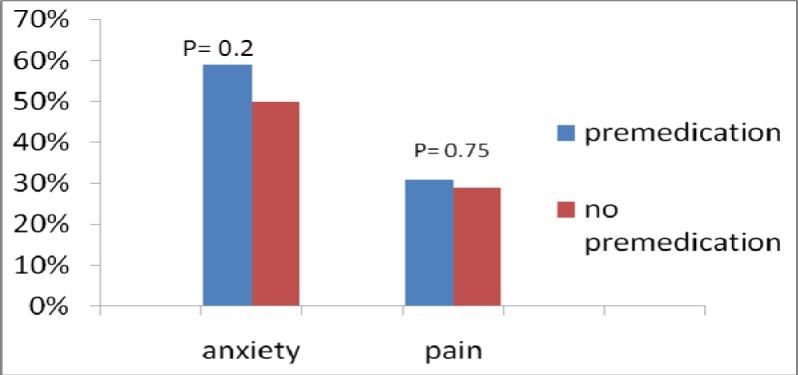
Effect of premedication on anxiety and pain perception.

A total of 200 consecutive patients with similar demographic characteristics (Table 1) were randomized into two groups (100 patients per group). The procedure was elective in more than 90% of the cases. The first group received open-label oral diazepam (5 mg) and chlorphenamine (4 mg) 60 minutes prior to their procedures, and the other group did not receive any premedication. The patient population was predominantly male (76%), with a mean age of 58 years. There were no differences in periprocedural pain perception (31% in the premedicated group versus 29% in the non-premedicated group; P = 0.75) or anxiety (59% in the premedicated group versus 50% in the non-premedicated group; P = 0.2; Fig. 1). Interestingly, local pain was more pronounced in the pre-medicated patients than in the non-premedicated patients (30% versus 16%; P = 0.018). Additional intravenous sedation with midazolam (2 mg) was required in 2% of the patients without premedication (Table 2).

**Table 1:** Baseline Characteristics of Patients

Variable	Premedication (n = 100)	No Premedication (n =100)	P-value
Male gender	74 (74%)	78 (78%)	0.5
Age (years)	57.9 ± 11	58.8 ± 12	0.6
Weight (kg)	79.3 ± 14.7	80.3 ± 15.2	0.6
FH of the IHD	41 (41%)	53 (53%)	0.09
Elective	91 (96%)	92 (92%)	0.28
Dyslipidemia	59 (59%)	65 (65%)	0.38
HTN	64 (64%)	64 (64%)	1
DM	50 (50%)	59 (59%)	0.2
Smoking	12 (12%)	12 (12%)	1
Presence of CAD	48 (48%)	44 (44%)	0.57

**Table 2:** Outcome Comparison Between the Premedicated and Non-premedicated Groups

Variable	Premedication n = 100 (%)	No Premedication n = 100 (%)	P-value
Pain during the procedure	31 (31)	29 (29)	0.75
Anxiety before the procedure	26 (26)	24 (24)	0.74
Anxiety after the procedure	6 (6)	7 (7)	0.77
Overall anxiety	59 (59)	50 (50)	0.2
Preexisting psychological illness	2 (2)	2 (2)	NS
Local pain	30 (30)	16 (16)	0.018
Comfortable during the procedure	81 (81)	81 (81)	NS
Intravenous sedation during the procedure	0	2 (2)	0.26
Catheter-induced spasms	4 (4)	0 (0)	0.04
Non-catheter-induced spasms	1 (1)	1 (1)	NS

Coronary spasms were observed in 0.03% of the patients, predominantly in the premedication group (0.05% versus 0.01%), and catheter-induced coronary spasms were more commonly observed than non-catheter-induced spasms (0.4% versus 0%, P = 0.04). There was no gender deference in coronary spasm (Table 3).

**Table 3:** The Relation Between the Gender and Coronary Spasm

	Male (%)	Female (%)	P-value
Catheter-induced spasms	3 (0.015)	1 (0.005)	NS
Non-catheter-induced spasms	1 (0.005)	1 (0.005)	NS

None of our patients experienced clinically significant adverse reactions or contrast allergies.

## Discussion

Premedication with diazepam and chlorphenamine is frequently used in the cardiac catheterization laboratory during coronary angiography and interventions. Nevertheless, data regarding their usefulness and efficacy are scarce. There is no standard premedication, and sedatives may be required in some patients to relieve their anxiety.

Conscious sedation is practiced at some institutions where the sedation protocol varies according to the institution’s guidelines [4]. Routine oral sedation as a premedication before cardiac catheterization in adults is now obsolete and lacks evidence-based practices. Certain interventional procedures (such as valvular procedures) may require sedation using intravenous medications. Evidence-based guidelines now exist, and patients receive intravenous midazolam in the cardiac catheterization laboratory in which the procedure takes place [5, 6].

All of the patients received appropriate pain relief medication following the procedure.

As in other studies, premedication with oral diazepam had little impact on the patients’ anxiety and pain perception [6]. Furthermore, oral diazepam and chlorphenamine did not reduce the number or occurrence of adverse effects following coronary angiography.

We found that the patients who received premedication reported a higher rate of experiencing local pain.

The findings of this study do not support the routine use of oral diazepam and chlorphenamine as premedications for patients undergoing coronary angiography and interventions.

The main finding of this study was that premedication does not reduce patient anxiety or pain perception, in contrast to Woodhead et al., who observed significant pain relief in diazepam-premedicated patients [1].

We examined the occurrence of non-catheter-induced coronary spasms with the use of premedications and compared our results to the observations by Bennett, who reported a higher incidence of spasms with sedation. Our study did not identify an increase in the number of non-catheter-related coronary spasms. In contrast, the number of catheter-induced spasms was significantly higher with premedication 4% versus 0% [2].

### Conclusion

Treatment with oral diazepam and chlorphenamine prior to cardiac catheterization and percutaneous coronary intervention did not alter the rates of anxiety, periprocedural pain, or contrast reactions. Premedication was associated with a higher incidence of catheter-induced coronary spasms.
